# Risk Factors Associated with *Stenotrophomonas maltophilia* Bacteremia: A Matched Case-Control Study

**DOI:** 10.1371/journal.pone.0133731

**Published:** 2015-07-24

**Authors:** Kosuke Sumida, Yong Chong, Noriko Miyake, Tomohiko Akahoshi, Mitsuhiro Yasuda, Nobuyuki Shimono, Shinji Shimoda, Yoshihiko Maehara, Koichi Akashi

**Affiliations:** 1 Emergency and critical care center, Kyushu University Hospital, Fukuoka, Japan; 2 Medicine and Biosystemic Science, Kyushu University Graduate School of Medical Sciences, Fukuoka, Japan; 3 Center for the Study of Global Infection, Kyushu University Hospital, Fukuoka, Japan; Bambino Gesù Children's Hospital, ITALY

## Abstract

*Stenotrophomonas maltophilia* is an important nosocomial bacterial pathogen, as is *Pseudomonas aeruginosa*. Differentiation of these bacteria as bacteremic agents is critical in the clinical setting and to define a therapeutic strategy; however, the associated factors and prognosis for *S*. *maltophilia* bacteremia have not been fully evaluated to adequately characterize these factors. We first conducted a matched case-control study to clarify these questions. A total of 30 case patients with *S*. *maltophilia* bacteremia were compared with 30 control patients with *P*. *aeruginosa* bacteremia between January 2005 and August 2014, according to matching criteria based on underlying disease, age, and gender. The 30-day mortality rate for the case patients (53.3%) was significantly higher than that of the control group (30.0%) (*P* = 0.047, using the log-rank test). Conditional logistic regression analysis showed that the predisposing factors specific for the detection of *S*. *maltophilia* bacteremia were indwelling artificial products other than a central venous catheter, ICU stay, and previous use of anti-MRSA drugs. The high severity of illness was associated with mortality in both case and control patients. Interestingly, inappropriate antimicrobial treatment was an additional independent risk factor for mortality in only the case patients with *S*. *maltophilia* bacteremia (odds ratio = 13.64, *P* = 0.048). Monotherapy with fluoroquinolones inactive against the *S*. *maltophilia *isolates was mainly responsible for the inappropriate treatment. These results suggest that more precise prediction and more appropriate treatment might improve the prognosis of patients with *S*. *maltophilia* bacteremia.

## Introduction

*Stenotrophomonas maltophilia* has emerged as an important pathogen that induces nosocomial infections [1]. *S*. *maltophilia* is a non-fermentative, gram-negative bacillus as well as *Pseudomonas aeruginosa* and causes severe infectious diseases, particularly bacteremia in the hospital setting, similar to those caused by *P*. *aeruginosa* [2,3]. However, the therapeutic strategy for the management of *S*. *maltophilia* is very different from that applied to *P*. *aeruginosa*, because *S*. *maltophilia* is intrinsically resistant to lots of antibiotics, including β-lactams and aminoglycosides [1]. Therefore, in a clinical setting that predisposes patients to *S*. *maltophilia* bacteremia, the differentiation between these two causative agents is critical.

A limited number of case-control studies on *S*. *maltophilia* bacteremia have been reported [4–10]. Various risk factors and mortality rates were obtained using different control groups [4–8]. Moreover, few studies have investigated *P*. *aeruginosa* bacteremia in control groups [9,10]. Adequate matching eliminates the influence of potentially confounding factors for underlying diseases and, in our study, it helped to more practically characterize the predisposing factors for *S*. *maltophilia* bacteremia and its prognosis in clinical settings similar to those that favor *P*. *aeruginosa* bacteremia. Therefore, we first conducted a matched case-control study using control patients with *P*. *aeruginosa* bacteremia. The risk factors for mortality of patients with bacteremia were also evaluated.

## Methods

### Patients and Ethics Statement

This matched, retrospective, case-control study was conducted at the Kyushu University Hospital, a 1,275-bed tertiary-care hospital in Fukuoka, Japan. Data were collected between January 2005 and August 2014 from electric medical records. First, patients who had positive blood culture for *S*. *maltophilia* or *P*. *aeruginosa* were selected. The Research Ethics Committee of Kyushu University Hospital approved this study under protocol No. 26–288 and exempted the need for obtaining informed consent from each patient.

### Enrollment and Matching Criteria

The analysis of bacteremia data indicated bacteremia due to *S*. *maltophilia* in 32 patients and due to *P*. *aeruginosa* in 122 patients. No blood samples were simultaneously positive for *S*. *maltophilia* and *P*. *aeruginosa*. In 2 patients, *S*. *maltophilia* was isolated from a single blood culture, and in subsequent blood culture, bacteremia was cleared without any treatment. These patients were excluded because of possible sample contamination and the remaining 30 patients with *S*. *maltophilia* bacteremia were enrolled as the case group. Control patients with *P*. *aeruginosa* bacteremia were matched to case patients on a 1:1 ratio using a stepwise procedure in the order of underlying disease, age, and gender, to ensure the best match. We focused on matching case and control patients with the same underlying primary disease. Subsequently, control patients with similar age (±10 years) and the same gender were chosen as case patients. If this criterion was not satisfied, control patients with an age similar to that of case patients were prioritized. Gender could not be matched in 3 patient pairs. All case and control patient pairs were matched with the same primary disease, as shown in Table 1. Patients with non-hematological diseases comprised 3 with cholangiocarcinoma, 1 with hepatocellular carcinoma, 4 with liver cirrhosis, 2 with dilated cardiomyopathy, and 4 with other diseases. Patients with the other non-hematological diseases included 2 with acute pancreatitis, 1 with acute pneumonia, and 1 with multiple trauma.

**Table 1:** Clinical characteristics of patients with *Stenotrophomonas maltophilia* or *Pseudomonas aeruginosa* bacteremia.

Characteristic	*S*. *maltophilia*	*P*. *aeruginosa*	*P*
Total no. of patients	30	30	
Gender, male	18 (60.0)	21 (70.0)	0.589
Age, mean years	51	52	0.762
Underlying disease			
hematological disease			
acute leukemia	10 (33.3)	10 (33.3)	NS
malignant lymphoma	5 (16.7)	5 (16.7)	NS
others	1 (3.3)	1 (3.3)	NS
non-hematological disease			
solid tumor	4 (13.3)	4 (13.3)	NS
hepatic disease	4 (13.3)	4 (13.3)	NS
heart disease	2 (6.7)	2 (6.7)	NS
others	4 (13.3)	4 (13.3)	NS

Data are no. (%) of patients, unless indicated otheriwse.

NS, not significant

### Variables and Definitions

Clinical data were collected from medical records to evaluate the risk factors and mortality rates. The variables associated with the detection of *S*. *maltophilia* or *P*. *aeruginosa* bacteremia included indwelling central venous catheter (CVC) or other artificial products, neutropenia, persistent neutropenia, prolonged hospitalization >30 days, ICU stay, history of chemotherapy and/or transplantation within 30 days, and previous antimicrobial treatment within 30 days. Artificial products other than a CVC primarily included endotracheal tube, drainage tube, urethral catheter and intravascular catheter other than a CVC. In many patients, more than two artificial products, such as an endotracheal tube and a urethral catheter, were simultaneously implanted such that we could not separately evaluate those factors by statistical analysis. Therefore, “artificial products” were used as a variable in the logistic analysis. Neutropenia was defined as the absolute neutrophil count of < 100 cells/mm^3^ at the onset of bacteremia [9]. Persistent neutropenia was defined as clinical episodes in which neutrophil counts <100/mm^3^ persisted for more than 2 weeks before the onset of bacteremia. The variables associated with the mortality of patients with *S*. *maltophilia* or *P*. *aeruginosa* bacteremia included neutropenia, polymicrobial bacteremia, septic shock [11], Sequential Organ Failure Assessment (SOFA) score [12], history of chemotherapy and/or transplantation within 30 days, and inappropriate antimicrobial treatment. Appropriate antimicrobial treatment was defined as a regimen that included 1 or more antimicrobial agent to which the isolate was susceptible in vitro [6], while those who received inappropriate treatment were indicated as all patients who were not included in the definition of appropriate treatment.

### Microbiological Analysis

After bacterial isolation using an automated blood culture system, the species were identified using the Vitek system (bioMerieux Japan Ltd., Tokyo, Japan). Antibiotic susceptibility was determined using the breakpoints standardized by the Clinical and Laboratory Standards Institute (CLSI) [13].

### Statistical Analysis

Gender and age were compared between the case and control groups using the chi-square test and the Mann-Whitney *U* test, respectively. Univariate and multivariate conditional logistic regression analyses were performed to extract risk factors for the detection of *S*. *maltophilia* bacteremia. The variables with a *P* < 0.2 on univariate analysis were included into the multivariate model. Univariate and multivariate logistic regression analyses were performed to assess the risk factors for mortality. Multivariate analysis was performed using a stepwise logistic regression model with a p-value cut-off point of 0.2. The log-rank test was used to compare the survival curves obtained using the Kaplan-Meier method for patients with *S*. *maltophilia* and *P*. *aeruginosa* bacteremia. *P* < 0.05 was considered to be statistically significant. All statistical analyses were performed using the JMP Pro software, version 11 (SAS Institute, Inc., Cary, NC, USA).

## Results

A total of 30 case patients with *S*. *maltophilia* bacteremia were matched with 30 control patients with *P*. *aeruginosa* bacteremia for underlying disease, age, and gender (Table 1). Approximately 50% of the patients with *S*. *maltophilia* bacteremia had hematological diseases. The 30-day mortality rate for the case and control patients was 53.3% (16/30 patients) and 30% (9/30), respectively, and this difference was statistically significant (*P* = 0.047). Therefore, the attributable mortality was 23.3%.

Case patients were compared with control patients considering the predisposing risk factors for the detection of *S*. *maltophilia* bacteremia using conditional univariate logistic analysis (Table 2). The frequency of CVC use was similar between the two groups whereas the other artificial products were more likely to be implanted in the case patients compared with the control patients (*P* = 0.016). In addition, multiple artificial products other than a CVC were present in 11 case patients and in 3 control patients. Moreover, in 7 of the 11 case patients, an endotracheal tube and a urethral catheter were used simultaneously. Thus, there was no specific trend in use of the artificial products. The mean duration of hospitalization before the onset of bacteremia was similar between the case and control patients. The case patients stayed in the ICU at the onset of bacteremia longer than the control patients (*P* = 0.038). Most of the case and control patients received antimicrobial therapy within 30 days of the onset of bacteremia. The use of carbapenems or antipseudomonal cephalosporins was not associated with the emergence of *S*. *maltophilia* bacteremia, while the frequency of patients who had taken anti-MRSA drugs was significantly higher among the case group compared with the control group. The multivariate analysis did not extract any independent risk factors for the detection of *S*. *maltophilia* bacteremia when compared with the matched control group with *P*. *aeruginosa* bacteremia.

**Table 2:** Univariate conditional logistic regression analysis of risk factors for *Stenotrophomonas maltophilia* bacteremia compared to *Pseudomonas aeruginosa* bacteremia.

	*S*. *maltophilia*	*P*. *aeruginosa*	OR (95%CI)	*P*
Variable	(n = 30)	(n = 30)		
Central venous catheter	22 (73.3)	21 (70.0)	1.25 (0.33–5.05)	0.739
Artificial products other than CVC	22 (73.3)	11 (36.7)	4.67 (1.52–20.24)	0.016
Neutropenia^*a*^	12 (40.0)	15 (50.0)	0.25 (0.01–1.69)	0.215
Persistent neutropenia^*b*^	9 (30.0)	5 (16.7)	2.33 (0.65–10.83)	0.22
Prolonged hospitalization of > 30 days	21 (70.0)	17 (56.7)	1.8 (0.62–5.86)	0.292
ICU stay before infection	11 (36.7)	3 (10.0)	5.0 (1.32–32.53)	0.038
Chemotherapy within 30 days	11 (36.7)	12 (40.0)	0.8 (0.20–3.02)	0.739
Transplantation within 30 days	8 (26.7)	5 (16.7)	1.75 (0.53–6.68)	0.372
Previous antimicrobial therapy				
Carbapenems	22 (73.3)	16 (53.3)	2.2 (0.80–6.98)	0.144
Anti-MRSAs^*c*^	19 (63.3)	7 (23.3)	3.4 (1.34–10.34)	0.016
Antipseudomonal cephalosporins	11 (36.7)	6 (20.0)	2.67 (0.77–12.17)	0.148
Fluoroquinolones	12 (40.0)	8 (26.7)	1.67 (0.62–4.90)	0.323
Aminoglycosides	5 (16.7)	5 (16.7)	1.0 (0.19–5.40)	1
TMP-SMX	10 (33.3)	14 (46.7)	0.5 (0.13–1.59)	0.258
Minocycline	4 (13.3)	2 (6.7)	2.0 (0.39–14.42)	0.424

Data are no. (%) of patients, unless indicated otheriwse.

^*a*^Neutropenia was defined as an absolute neutrophil count of < 100 cells/mm^3^ at the onset of bacteremia.

^*b*^Persistent neutropenia was defined as an episode in which a neutrophil count <100/mm^3^ persisted more than 2 weeks before the onset.

^*c*^Anti-MRSAs included glycopeptides, linezolid, and daptomycin.

OR, odds ratio; CI, confidence interval; CVC, central venous catheter; ICU, intensive care unit; MRSA, methicillin-resistant Staphylococcus aureus; TMP-SMX, trimethoprim-sulfamethoxazole

Univariate logistic analysis was performed to assess the risk factors for mortality in the patients with *S*. *maltophilia* or *P*. *aeruginosa* bacteremia (Table 3). Severe neutropenia and chemotherapy or transplantation were not associated with the mortality of patients with *S*. *maltophilia* bacteremia. High severity score was a factor significantly associated with the mortality in the patients with *S*. *maltophilia* bacteremia as well as in those with *P*. *aeruginosa* bacteremia (*P* = 0.006 and 0.01, respectively). Inappropriate antimicrobial therapy was an additional risk factor for the mortality in patients with *S*. *maltophilia* bacteremia (*P* = 0.045). The results of the multivariate analysis also indicated that inappropriate antimicrobial therapy was an independent risk factor for mortality in patients with *S*. *maltophilia* bacteremia, as shown in Table 4 (odds ratio = 13.64, *P* = 0.048). The inappropriate antimicrobial treatment in the 7 non-surviving patients with *S*. *maltophilia* bacteremia included 3 patients who had not taken any agents recommended for *S*. *maltophilia* and 4 patients in whom monotherapy with fluoroquinolones was inactive against the isolated *S*. *maltophilia*. Specifically, patients with *S*. *maltophilia* bacteremia had monotherapy with fluoroquinolones in 6 of the 16 non-survivors and 3 of the 14 survivors. Among the 6 non-survivors, 4 patients received only fluoroquinolones, to which *S*. *maltophilia* were not susceptible.

**Table 3:** Univariate logistic regression analysis of risk factors for mortality in patients with *Stenotrophomonas maltophilia* or *Pseudomonas aeruginosa* bacteremia.

	Patients with *S*. *maltophilia* bacteremia				Patients with *P*. *aeruginosa* bacteremia			
Variable	Non-survivors	Survivors	OR (95%CI)	*P*	Non-survivors	Survivors	OR (95%CI)	*P*
	(n = 16)	(n = 14)			(n = 9)	(n = 21)		
Neutropenia^*a*^	8 (50.0)	4 (28.6)	2.5 (0.57–12.39)	0.237	4 (44.4)	11 (52.4)	0.73 (0.14–3.51)	0.691
Polymicrobial bacteremia	5 (31.3)	3 (21.4)	1.7 (0.33–9.80)	0.546	3 (33.3)	5 (23.8)	1.6 (0.26–8.86)	0.59
Septic shock	5 (31.3)	1 (7.1)	5.9 (0.79–122.45)	0.129	6 (66.7)	6 (28.6)	5.0 (0.99–30.64)	0.06
SOFA score >6	12 (75.0)	3 (21.4)	11.0 (2.22–71.64)	0.006	8 (88.9)	6 (28.6)	20.0 (2.82–415.32)	0.01
Chemotherapy within 30 days	7 (43.8)	5 (35.7)	1.4 (0.32–6.38)	0.654	3 (33.3)	9 (42.9)	0.67 (0.12–3.29)	0.627
Transplantation within 30 days	6 (37.5)	2 (14.3)	3.6 (0.66–28.50)	0.165	1 (11.1)	4 (19.0)	0.53 (0.02–4.37)	0.597
Inappropriate antimicrobial treatment	7 (43.8)	1 (7.1)	10.1 (1.45–206.61)	0.045	2 (22.2)	1 (4.8)	5.71 (0.48–134.76)	0.18

Data are no. (%) of patients, unless indicated otheriwse.

^*a*^Neutropenia was defined as an absolute neutrophil count of < 100 cells/mm^3^ at the onset of bacteremia.

OR, odds ratio; CI, confidence interval; SOFA, Sequential Organ Failure Assessment

**Table 4:** Independent risk factors for mortality in patients with *Stenotrophomonas maltophilia* or *Pseudomonas aeruginosa* bacteremia.

	Patients with *S*. *maltophilia* bacteremia		Patients with *P*. *aeruginosa* bacteremia	
Variable	OR (95%CI)	*P*	OR (95%CI)	*P*
SOFA score >6	13.65 (2.27–128.44)	0.009	20.0 (2.82–415.32)	0.01
Inappropriate antimicrobial treatment	13.64 (1.40–355.43)	0.048	-	-

OR, odds ratio; CI, confidence interval; SOFA, Sequential Organ Failure Assessment

## Discussion

It is possible to assume that the clinical settings are similar between the hospitalized patients with *S*. *maltophilia* and *P*. *aeruginosa* bacteremia. Therefore, to adequately characterize the associated factors and mortality rates for *S*. *maltophilia* bacteremia, matching patients with *S*. *maltophilia* bacteremia with those with *P*. *aeruginosa* bacteremia is important. The clinical setting used in the present matched case-control study was similar for both patient groups.

The number of case-control studies for *S*. *maltophilia* bacteremia is limited. Moreover, since the control patients involved not only those with *P*. *aeruginosa* bacteremia, but also those with *Escherichia coli* bacteremia or those without bacteremia, the extraction of different risk factors for *S*. *maltophilia* bacteremia was inevitable. The presence of CVC was not associated with the occurrence of *S*. *maltophilia* bacteremia in this study. In previous studies, CVC was a factor associated with *E*. *coli* bacteremia [4,8], but not with *P*. *aeruginosa* bacteremia [9,10]. These findings clearly indicate that the background of control patients influences data extraction on the risk factors for the case patients. Interestingly, indwelling artificial products other than a CVC and ICU stay were associated with *S*. *maltophilia* bacteremia, although these factors were not independent. In fact, all of the ICU patients placed not only a CVC, but also multiple artificial products. ICU stay and the implantation of multiple artificial products more likely induce the emergence of *S*. *maltophilia* bacteremia even when compared with *P*. *aeruginosa* bacteremia.

Previous treatment with antipseudomonal broad-spectrum antibiotics has been reported to be a risk factor for the detection of bacteremic *S*. *maltophilia* [6,8,10]. Our results indicated that the use of carbapenems or antipseudomonal cephalosporins was not a factor that predisposed *S*. *maltophilia* bacteremia. This result may be attributable to having organized patients’ medical history using the matching process conducted in our study. Thus, it may be difficult to distinguish bacteremia due to *S*. *maltophilia* from bacteremia due to *P*. *aeruginosa* based on the previous use of these agents. On the other hand, the use of anti-MRSA agents was associated with *S*. *maltophilia* bacteremia in univariate analysis, which is consistent with the results of other studies [8,10]. It is difficult to adequately interpret this finding because anti-MRSA agents can induce the colonization of both of *P*. *aeruginosa* and *S*. *maltophilia*. Further studies are needed to elucidate this issue.

It has been reported that the severity of illness is associated with poor outcome among patients with *S*. *maltophilia* bacteremia [9,10,14,15], which was corroborated by the results of the present study. Patients with hematological malignancies accounted for the majority of those with *S*. *maltophilia* bacteremia, with a frequency ranging between 40% and 60% [6,14,16,17], and this rate was approximately 50% in our hospital. The presence of neutropenia and/or transplantation, which is often associated with hematological malignancies, has been reported to be a risk factor for the mortality of patients with *S*. *maltophilia* bacteremia [6,9,14,17,18], but these factors were not significant in this study. Rather, inappropriate antimicrobial treatment was associated with the mortality of patients with *S*. *maltophilia* bacteremia. The results of the univariate analysis indicated that inappropriate therapy led to poor outcome following *S*. *maltophilia* bacteremia [6,9,16,19]. In this study, this parameter was extracted as an independent factor in the multivariate analysis.

Recent studies indicated that fluoroquinolones are a feasible alternative to trimethoprim-sulfamethoxazole (TMP-SMX) for the treatment of *S*. *maltophilia* bacteremia [20,21]. In our study, approximately 70% of the patients were treated with quinolones. However, the rapid acquisition of resistance to quinolones has been a cause for concern [1,22]. In this study, monotherapy with quinolones inactive against the isolated *S*. *maltophilia* had a negative impact on prognosis. The effectiveness of quinolone monotherapy against *S*. *maltophilia* bacteremia should be carefully assessed. Even though TMP-SMX plays a central role in the treatment of *S*. *maltophilia* infection [1,2], only 20% of the patients in this study were treated with that agent. In addition, the use rate of TMP-SMX was low regardless of the prophylactic treatment prior to the onset of bacteremia. The use of TMP-SMX may need to be better evaluated in our hospital.

The most important limitation of the present study involved the sample size, which especially, might be small to adequately assess the prognostic factors for *S*. *maltophilia* bacteremia. Another limitation of this study was data collection at a single institution. The choice of therapeutic agents for *S*. *maltophilia* bacteremia may tend to be different at each institution. Thus, inappropriate treatment could not be a risk factor for mortality in other hospitals. The two groups do not match according to SOFA score. It may be considered a limitation of this study because it is not possible to assess that the attributable mortality was not influenced by the severity of the underlying disease.

This is the first case-control study of patients with *S*. *maltophilia* bacteremia matched with a control group with *P*. *aeruginosa* bacteremia. The multivariate analysis applied to the matched pairs did not extract any independent factors for the detection of *S*. *maltophilia* bacteremia. This result indicates that we cannot easily diagnose the emergence of either *S*. *maltophilia* or *P*. *aeruginosa* in cases of bacteremia. However, the univariate analysis possibly indicates that further analysis may help extract data on the use of artificial products or ICU stay as independent factors for *S*. *maltophilia* bacteremia. Moreover, this study suggested that the high mortality due to *S*. *maltophilia* bacteremia was not attributable to neutropenia or transplantation in patients with hematological malignancies, but rather to inappropriate antimicrobial treatment. Therefore, the prognosis of patients with *S*. *maltophilia* bacteremia may be improved, at least to the same level as that of those with *P*. *aeruginosa* bacteremia, through adequate antimicrobial therapy.
